# Small Vessel Disease: Another Component of the Hypertrophic Cardiomyopathy Phenotype Not Necessarily Associated with Fibrosis

**DOI:** 10.3390/jcm10040575

**Published:** 2021-02-04

**Authors:** Monica De Gaspari, Cristina Basso, Martina Perazzolo Marra, Stefania Elia, Maria Bueno Marinas, Annalisa Angelini, Gaetano Thiene, Stefania Rizzo

**Affiliations:** Cardiovascular Pathology Unit and Cardiology Unit, Azienda Ospedaliera, Department of Cardiac, Thoracic, Vascular Sciences and Public Health, University of Padua Medical School, Via A. Gabelli, 61, 35121 Padova, Italy; monica.deg1@gmail.com (M.D.G.); martina.perazzolomarra@unipd.it (M.P.M.); stefania.e.pd@gmail.com (S.E.); maria.bueno.m@gmail.com (M.B.M.); annalisa.angelini@unipd.it (A.A.); gaetano.thiene@unipd.it (G.T.); s.rizzo@unipd.it (S.R.)

**Keywords:** hypertrophic cardiomyopathy, pathology, small vessel disease

## Abstract

Background: Hypertrophic cardiomyopathy (HCM) is characterized by myocardial disarray, small vessel disease (SVD), and fibrosis. The relationship between SVD and replacement-type fibrosis is still unclear. Methods: Histopathologic assessment of replacement-type fibrosis and SVD in HCM patients with either end-stage heart failure (HF) or sudden cardiac death (SCD). Chronic ischemic heart disease (IHD) patients served as controls. Results: Forty HCM hearts, 10 HF and 30 SCD, were studied. Replacement-type fibrosis was detected in all HF and in 57% of SCD cases. In SCD, replacement-type fibrosis was associated with older age, greater septal thickness, SVD prevalence, and score (all *p* < 0.05). Prevalence of SVD did not show significant differences among SCD, HF, and IHD (73%, 100% and 95%, respectively), while SVD score was higher in HF than IHD and SCD (2.4, 1.95, and 1.18, respectively) and in areas with replacement-type fibrosis vs. those without in HF (3.4 vs. 1.4) and SCD (1.4 vs. 0.8) (all *p* < 0.05). Conclusions: SVD is a frequent feature in HCM independent of the clinical presentation. A higher SVD score is observed in HCM-HF and in areas with replacement-type fibrosis. Although SVD is part of the HCM phenotype, further remodeling of the microcirculation might occur secondarily to fibrosis.

## 1. Introduction

Hypertrophic cardiomyopathy (HCM) is an inherited myocardial disease due to mutations of genes that mostly encode for sarcomeric proteins, with a heterogeneous clinical and prognostic spectrum [1]. It is characterized by either symmetric or asymmetric hypertrophy, involving predominantly the interventricular septum (IVS) which can cause left ventricular (LV) outflow tract obstruction [1,2]. Histological abnormalities affect not only the cardiac myocyte and the interstitial space, with hypertrophy, disarray, and both interstitial and replacement-type fibrosis, but also the microcirculation, with thickening of the walls of the intramyocardial arterioles. These myocardial features may represent the substrates of either ventricular arrhythmias at risk of sudden cardiac death (SCD) or evolution towards systolic dysfunction (so-called “end-stage” heart failure—HF) [3,4,5,6,7].

While the clinical and prognostic impact of microvascular impairment has been demonstrated by positron emission tomography (PET) and cardiac magnetic resonance (CMR) studies in HCM, the pathogenesis of small vessel disease (SVD) in HCM is not yet known and the relationship between SVD and replacement-type fibrosis is still debated.

Our aim was to evaluate replacement-type fibrosis and SVD in HCM hearts coming from patients with either HF or SCD, and in particular, the distribution of replacement-type fibrosis and the extent and type of SVD. The relationship of SVD to replacement-type fibrosis was then assessed in control hearts with chronic ischemic heart disease (IHD), since in this setting, association of SVD with fibrosis is a well-described finding.

## 2. Material and Methods

### 2.1. Study Population

We retrospectively reviewed formalin-fixed whole hearts with a pathologic diagnosis of HCM coming from patients with HF and undergoing heart transplantation (HT) or presenting with SCD, referred to the Cardiovascular Pathology Unit—Regional Registry of Cardio-cerebro-vascular Pathology, University Hospital of Padua. Samples were anonymous to the investigators.

HCM is defined as an LV hypertrophy (wall thickness ≥15 mm or equivalent for infancy and childhood), either symmetric or asymmetric (IVS to LV free wall thickness ratio ≥1.3), in the absence of aortic stenosis or systemic hypertension [2].

The study population consisted of three groups:

(a) Autopsy hearts of patients with SCD due to HCM (<40 years);
(b) Explanted native hearts of patients who underwent HT due to end-stage HF-HCM;
(c) Autopsy hearts of patients with post-myocardial infarction chronic IHD who died due to HF.

For HCM hearts, exclusion criteria were the presence of a myocardial bridge or of critical coronary artery disease (luminal stenosis >70%) affecting a major epicardial vessel.

### 2.2. Gross Examination

The following features have been evaluated for each cardiac specimen according to the current guidelines [8]:

- Total heart weight, paying attention to removal of pericardium, postmortem clots, and cutting the great arteries 2 cm above semilunar valves, venae cavae, and pulmonary veins at their junctions with the atria in case of autopsy.
- Wall thickness of the left ventricle (LV) free wall, IVS and right ventricle (RV) free wall, to assess the subtype of hypertrophy (symmetric or asymmetric), in cross-section, excluding papillary muscles and trabeculae [5,8]; normal heart weight and wall thickness for sex and age were calculated according to Schulz and Giordano [9] for children and to Kitzman et al. [10] for adults.
- Coronary artery in terms of origin, course, in particular the presence of myocardial bridge at the level of left anterior descending artery, and patency with serial transverse cuts.
- Presence and site of grossly evident myocardial scars.

For each heart, a total transverse section including LV, IVS, and RV was sampled at the mid-ventricular level.

### 2.3. Histological Analysis

All tissue specimens were embedded in paraffin, sectioned at 5-mm thickness, and stained with hematoxylin and eosin, Heidenhain trichrome, and Weigert van Gieson.

The following parameters were evaluated in each case: cardiomyocyte hypertrophy (myocytes enlarged with hyperchromatic nuclei and diameters greater than 20 µm), myocardial disarray, myocyte vacuolization (myocytolysis), replacement-type fibrosis (presence, distribution subendocardial, mid-mural, subepicardial, transmural—and extent).

**Figure 1:**
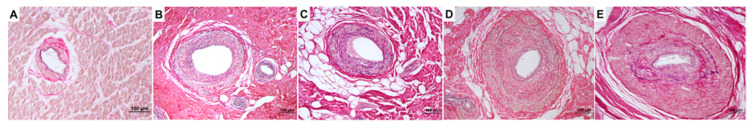
Score of small vessel disease based upon luminal narrowing ((**A**), 0, no abnormality; (**B**), 1+, luminal stenosis <20%; (**C**), 2+, luminal stenosis 20–50%; (**D**), 3+, luminal stenosis >50–90%; (**E**), 4+, luminal stenosis >90%). ((**A**–**E**), Weigert van Gieson).

Intramyocardial SVD (arterioles diameter 100–500 μm) was qualitatively (i.e., intimal hyperplasia or medial hypertrophy/fibrosis) and semi-quantitatively assessed in cross-section. A scoring system according to the degree of luminal narrowing was used as follows: 0 (no abnormality), 1+ (<20%), 2+ (20–50%), 3+ (>50–90%), 4+ (>90%) (Figure 1). The highest observed score for each tissue section was noted. The analysis was performed for each heart specimen on two sections at the level of the lateral LV free wall and mid IVS. Moreover, we studied the association of SVD with areas of replacement-type fibrosis.

### 2.4. Statistical Analysis

Data are expressed as mean ± standard deviation and median with 95% CI. Differences between continuous variables were assessed with unpaired Student’s t-test and Mann–Whitney test. A Kruskal–Wallis test was applied when comparing more than 2 groups. It is used for multiple comparisons of three or more independent samples when they present non-parametric distribution. Proportions were compared by Chi-square or Fisher’s exact tests. Correlation analysis between selected variables was assessed by Spearman’s rank-order correlation coefficient. A *p* ≤ 0.05 was considered significant.

## 3. Results

**Figure 2:**
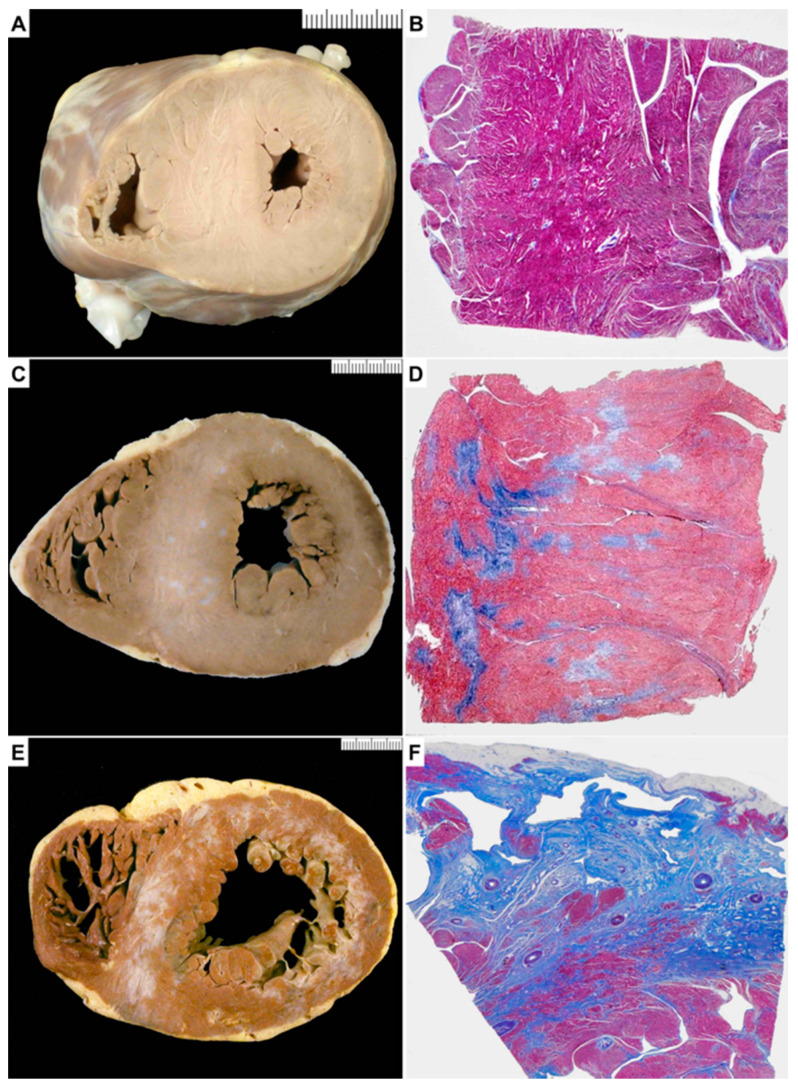
Gross and histological analysis of HCM hearts with different clinicopathologic phenotypes. (**A**,**B**) SCD-HCM patient with massive septal hypertrophy, without evidence of replacement-type fibrosis both grossly and histologically. (**C**,**D**) SCD-HCM patient with grossly evident scars at the level of the IVS, confirmed by histology. (**E**,**F**) HF-HCM patient with thinning of the LV free wall and IVS due to widespread scarring, confirmed at histology as replacement-type fibrosis associated with small vessel remodeling. (**B**,**D**,**F**) Heidenhain trichrome, panoramic view.

Main gross and histologic findings are summarized in Table 1 and Figure 2.

### 3.1. Gross Examination

(a) SCD due to HCM (<40 years). Thirty consecutive hearts from young patients (<40 years, mean age 22.6 ± 7.4, range 7–38; 25 males (83%)) who died suddenly due to HCM were enrolled. Heart weight ranged from 220 to 1200 g (mean 538 ± 202.90 g) with a net rise of 95.30 ± 63.42% compared to the mean normal weight value for sex and age. The mean thickness of the IVS was 20.8 ± 7.14 mm, LV free wall 16.30 ± 4.04 mm, and RV free wall 4.52 ± 1.45 mm, with an increase compared to the mean normal thickness value for sex and age of 58.70 ± 54.65% in the IVS and 41.33 ± 53.77% in the LV free wall.

Hypertrophy was asymmetric in 21 cases (70%), septal in 17, and lateral in 4 cases.

Macroscopically evident myocardial scars were present in 10 cases (30%), with a mid-mural distribution in 7 cases (70%), subendocardial in 2 cases (20%), and transmural in 1 case (10%).

(b) Explanted native hearts of HCM patients with end-stage HF. Ten consecutive cases (mean age 51.60 ± 13.21, range 22–68; 4 males, 40%) who underwent HT for end-stage HF-HCM were enrolled. Heart weight ranged from 350 to 620 g (mean 425.70 ± 96.99 g) and with a mild rise of 44.40 ± 29.57% compared to the mean normal weight value for sex and age. The mean thickness of the IVS was 14.7 ± 3.09 mm, LV free wall 12.11 ± 2.02 mm, and RV free wall 4.77 ± 2.10 mm.

Hypertrophy was asymmetric septal in 5 cases (50%). Macroscopically evident myocardial scars were present in all (100%), mid-mural and transmural in 9 cases (90%), and subendocardial in 1 case (10%).

(c) Control group—chronic IHD. Twenty hearts from patients with post-myocardial infarction chronic IHD who died due to HF (mean age 60.6 ± 5.36, range 45–68 years, 19 males, 95%) were analyzed. Macroscopically evident transmural replacement-type fibrosis was present in all (100%).

### 3.2. Histological Analysis

Cardiomyocyte hypertrophy and myocardial disarray associated with interstitial-type fibrosis were observed in all HCM hearts, both SCD and HF. Moreover, focal myocytolysis as a sign of chronic ischemic damage was present.

Large areas of replacement-type fibrosis were present in all HF-HCM cases (100%), both in the lateral LV free wall and IVS, without statistically significant differences in terms of age (see Supplemental Figure S1). Replacement-type fibrosis was detected in 17 SCD-HCM cases (57%), with a significant difference compared to the HF group (*p* = 0.0164). In the SCD group, cases with replacement-type fibrosis showed significantly older age (*p* = 0.0432) and greater absolute heart weight (*p* = 0.0421) and absolute thickness of the IVS (*p* = 0.0023). In the SCD group, prevalence of the replacement-type fibrosis did not show a statistically significant difference when comparing asymmetric (12/21, 57%) vs. symmetric forms (4/9, 44%, *p* = NS). Within asymmetric cases, replacement-type fibrosis was more prevalent in the IVS (12/21, 57%) than in the lateral LV free wall (6/21, 28.5%, *p* = 0.01) (see Supplemental Figure S1).

Intramural arterioles were dysplastic and showed medial hypertrophy and intimal hyperplasia both in SCD and HF-HCM cases, in comparison with IHD characterized by medial hypertrophy and perivascular fibrosis.

**Figure 3:**
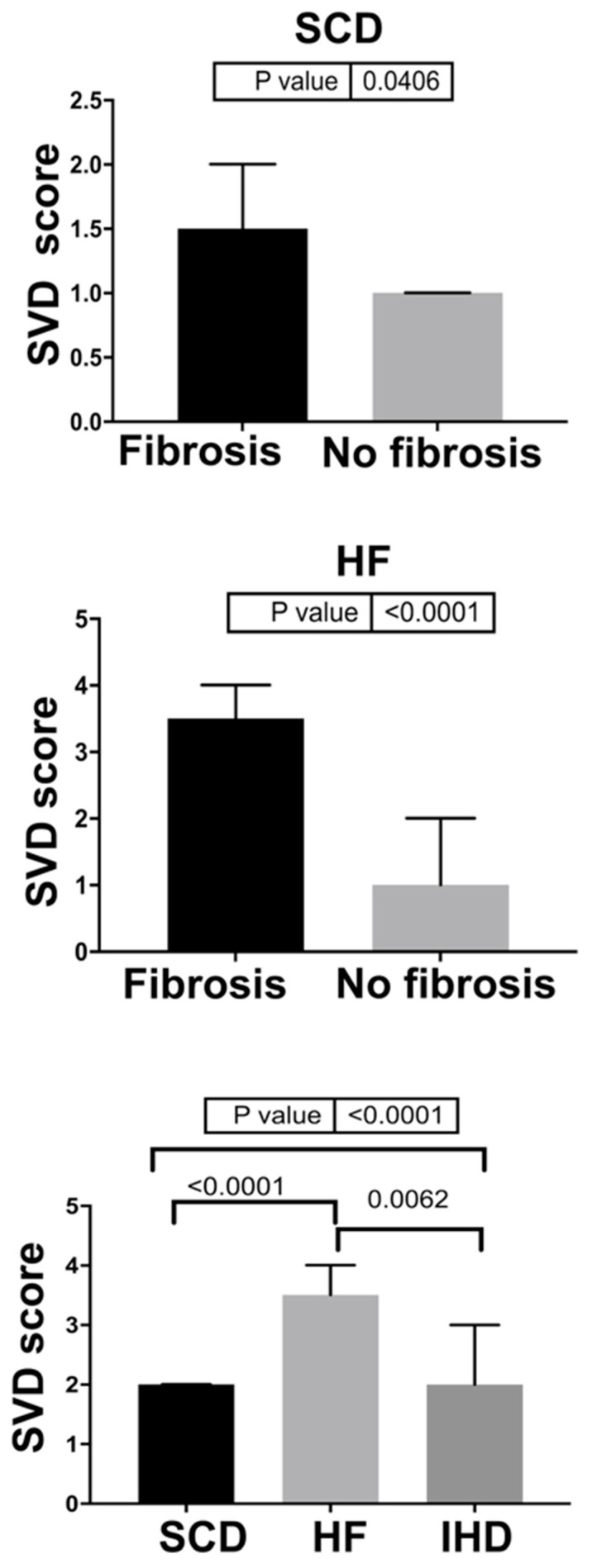
SVD score analyses in the study population. SCD-HCM with replacement-type fibrosis vs. without replacement-type fibrosis; HF-HCM with replacement-type fibrosis vs. without replacement-type fibrosis (*p* value calculated by Mann–Whitney test); SCD-HCM vs. HF-HCM vs. IHD (*p* values calculated by Kruskal–Wallis test).

Abnormal arterioles with luminal narrowing were detected in all HCM patients, both in fibrotic and non-fibrotic areas. The presence of SVD did not show significant differences among the groups (100% in the HF group, 73% in the SCD group, and 95% in the IHD group). A significant difference in SVD score was instead found between HF-HCM and SCD-HCM (*p* < 0.0001), HF-HCM and IHD (*p* = 0.0062), and SCD-HCM and IHD (*p* < 0.0001) (Figure 3).

In the SCD group, SVD was present in 6/9 (66.5%) of symmetric vs. 17/21 (81%) of asymmetric HCM; within the asymmetric HCM group, the prevalence of SVD was similar in the IVS (16/21, 76%) and in the lateral LV free wall (15/21, 71.5%, *p* = NS).

Finally, we assessed the overall HCM population, and then the HF-HCM group and the SCD-HCM group, separately, by comparing SVD score in females vs. males. No statistically significant difference was found considering patient gender (all *p* = NS, see Supplemental Figure S2).

**Figure 4:**
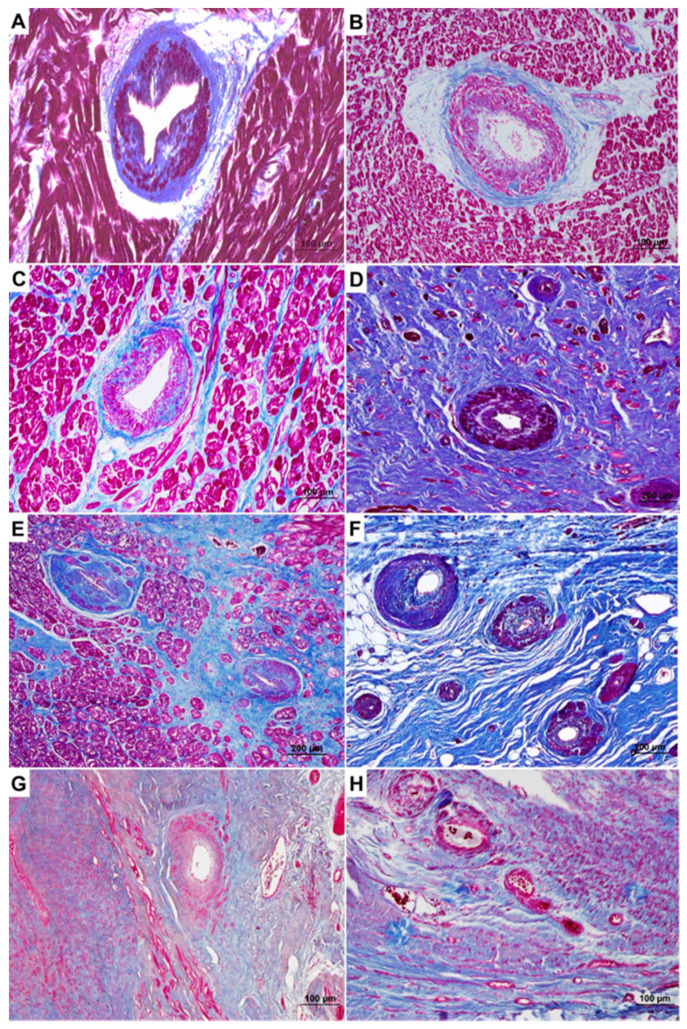
SVD in the study population: exemplificative histological features in the various subgroups. (**A**,**B**) SCD-HCM patient with SVD in the absence of replacement-type fibrosis. (**C**,**D**) SCD-HCM patient with SVD in areas without (**C**) and with (**D**) fibrosis: note the higher score in the area with replacement-type fibrosis. (**E**,**F**) HF-HCM patient with SVD in areas without (**E**) and with (**F**) fibrosis. (**G**,**H**) IHD patient showing large areas of replacement-type fibrosis with SVD, with a lower score as compared to HCM cases. (**A**–**H**) Heidenhain trichrome.

In chronic IHD, replacement-type fibrosis was typically associated with neo-vessels formation, characterized by hypertrophy of the tunica media and a mean SVD score of 1.95 ± 0.88. Noteworthy, SVD score was higher in areas with replacement-type fibrosis vs. areas without in both HF (3.4 vs. 1.4, respectively) and SCD (1.4 vs. 0.8, respectively) HCM hearts (all *p* < 0.05). No correlation was found between SVD score and age, net increase in heart weight and in IVS thickness in both SCD-HCM and HF-HCM cases (all *p* = NS, see Supplemental Table S1). The main histological findings are summarized in Table 1 and Figure 4.

## 4. Discussion

The present study was undertaken to evaluate the relationship between SVD and replacement-type fibrosis in HCM patients presenting with either SCD or end-stage systolic HF. Early studies in the literature described the classical pathological hallmarks of HCM, in terms of myocyte hypertrophy, disarray, interstitial and replacement-type fibrosis, and remodeling of the intramyocardial small vessels. We previously demonstrated that fibrosis in HCM is most probably ischemic in origin and represents the ideal substrate for electrical instability and life-threatening ventricular arrhythmias [5], so that CMR is now considered for risk stratification in HCM patients [11].

Moreover, extensive and transmural scarring leads to LV remodeling and end-stage HF, while no or only focal scars are present in patients with LV outflow tract obstruction and non-obstructive HCM with preserved systolic function [7].

In the current study, we confirm that the replacement-type fibrosis, always present in cases of HF-HCM, is absent in almost half of juvenile SCD cases. The presence of fibrosis is related to age, myocardial mass, and wall thickness in SCD-HCM.

Although SVD is present in both HCM groups, the highest SVD score is observed in HCM hearts with HF and in areas with replacement-type fibrosis vs. those without, thus confirming original observations [3,4].

It is well known that HCM patients may exhibit symptoms like angina in the absence of atherosclerosis of the major epicardial coronary vessels. Several mechanisms may contribute to ischemia and replacement-type fibrosis at risk of fatal arrhythmias in HCM, including demand–supply mismatch due to hypertrophy, reduced perfusion pressure related to shortened diastolic time, high diastolic pressure, LV outflow tract obstruction, myocardial bridging, and SVD [5,12]. Decreased myocardial perfusion by PET has been demonstrated in HCM, also in asymptomatic individuals, pointing to a coronary microcirculation impairment already at early stages of the disease [13,14]. Similar decrements in coronary resistance reserve were measured, implying that these findings could not be explained by increments in extravascular compression [15].

Maron et al. [3] first reported the abnormalities of the small intramyocardial arteries with increased wall thickness in the 83% of victims suffering from HCM at autopsy. The SVD has been then studied separately in SCD patients [5], in HT [6,16,17], and in surgical myectomy [6,18,19,20]. Typically, medial hypertrophy and/or intimal hyperplasia and dysplasia are observed, inducing lumen stenosis of small arteries. Moreover, pathological studies demonstrate reduced capillary supply [21].

The presence of SVD in all HCM cases, but with a higher score in areas of replacement-type fibrosis compared to those without, suggests that structural changes in the microcirculation are somehow a component of the morphological spectrum of HCM. Our findings confirm previous data by Foà et al. [6], who demonstrated that microvasculopathy is always present in both obstructive HCM and end-stage HCM. By reviewing the published studies addressing the pathology of SVD in HCM (Table 2), a relationship between SVD and fibrosis has been found in all but one [3,4,5,6,16,18,20]. However, we should stress that different methodologies to assess SVD have been used, ranging from quantitative to semiquantitative scores, with various definitions of intramyocardial vessel abnormalities; uniform methodology and terminology should be provided by pathologists.

Our data further demonstrate that the microcirculation can undergo an additional remodeling for multifactorial reasons in the areas of scarring, as it occurs in chronic post-infarct IHD in which, however, small vessels did not show any structural dysplasia and a lesser degree of narrowing. We cannot exclude the possibility that the occurrence of arteriolar changes in HCM is an independent phenomenon and not directly related to the degree of fibrosis. As in previous studies, no significant correlation between SVD and patient age has been found [3,16].

A significant difference in SVD scores in HF vs. SCD, HF vs. IHD, and SCD vs. IHD cases was instead found in our study. However, we did not find a significant association between SVD score and hypertrophy. By comparing HCM patients with controls, we observed that severe SVD is typical of HCM.

SVD also represents a common finding in animal models of HCM, most prevalent in sections with moderate or severe fibrosis and characterized by medial and intimal thickening associated with increased connective tissue elements [22,23]. Maron et al. [3] described the presence of SVD in the case of hypertrophy due to systemic hypertension and aortic valve stenosis, even if with a lower prevalence (9% vs. 83%). This finding suggests that although an LV overload might be involved in the pathogenesis of SVD as neo-angiogenesis of the fibrotic areas, it could be not the only factor in HCM. Structurally remodeled intramural coronary arterioles are distributed throughout hypertrophied and non-hypertrophied areas of the LV, either within or in close proximity to areas of replacement-type fibrosis [3]. A different type of arteriolar remodeling might indicate distinct pathophysiologic processes that may contribute to the ischemic injury in these two pathological conditions. In IHD, many neovessels in the scar, initially thin-walled, undergo a phase of vascular maturation and remodeling, leading to the progressive increase in the percentage of pericyte-coated microvessels and a rise in arteriolar density during cardiac repair. This process is induced by hypoxia and is associated with a release of angiogenic factors [24]. Furthermore, in hypertensive heart disease, perivascular fibrosis with increased arterial stiffness is a frequent finding and for this reason, we excluded cases with a history of hypertension in our series [25].

On the contrary, the pathogenesis of SVD in HCM, which has not been consistently reproduced in transgenic animal models of the disease, has not yet been determined. As recently emphasized, embryological factors have been claimed, as a response to a hypercontractile myogenic tube during development, with a possible impact on the microcirculation [26].

In the clinical setting, no specific treatment has been shown to significantly improve impaired microvascular function, probably underlying the crucial role of irreversible structural remodeling of the vessel wall. Thus, control of potential triggers of ischemia remains the most effective strategy.

In conclusion, replacement-type fibrosis is a constant feature in HCM patients with end-stage HF, while it is present in half of those with SCD. It correlates with age, cardiac mass, and wall thickness. The presence of SVD in all HCM cases, although with a higher score in those with replacement-type fibrosis, suggests that SVD represents a hallmark of HCM, probably leading to and worsened by fibrosis. These findings confirm that abnormal arterioles might contribute to the perfusion abnormalities found in HCM patients, resulting in recurrent myocardial ischemia and scarring. To better understand the relationship between fibrosis and SVD, follow-up perfusion and tissue characterization studies on a large series of HCM patients are warranted.

## Figures and Tables

**Table 1:** Study groups: main cardiac pathological findings.

	SCD Group (*n* = 30)	HF Group (*n* = 10)	IHD Group (*n* = 20)	*p* Value (SCD vs. HF)
Male, *n* (%)	25 (83)	4 (40)	19 (95)	0.006
Age, yrs (mean ± SD)	22.6 ± 7.4	51.60 ± 13.21	60.6 ± 5.36	<0.0001
Mean mass, g (mean ± SD)	538 ± 202.90	425.70 ± 96.99	446.39 ± 105.43	0.10
Thickness of the IVS, mm (mean ± SD)	20.8 ± 7.14	14.7 ± 3.09	11.8 ± 2.76	0.01
Thickness of the LVFW, mm (mean ± SD)	16.30 ± 4.04	12.11 ± 2.02	10.4 ± 2.35	0.008
Thickness of the RVFW, mm (mean ± SD)	4.52 ± 1.45	4.77 ± 2.10	3.8 ± 1.82	0.6
Gross fibrosis, *n* (%)	10 (30)	10 (100)	20 (100)	<0.0001
Histological replacement-type fibrosis, *n* (%)	17 (57)	10 (100)	20 (100)	0.01
SVD presence, *n* (%)	22 (73)	10 (100)	19 (95)	0.07
SVD score, mean	1.18	2.4	1.95	<0.0001

Abbreviations: SCD, sudden cardiac death; HF, heart failure; IHD, ischemic heart disease; SD, standard deviation; IVS, interventricular septum; LVFW, left ventricular free wall; RVFW, right ventricular free wall; SVD, small vessel disease.

**Table 2:** SVD and relationship with fibrosis in HCM: literature review.

Author, Year (Ref)	Source	HCM Cases, *n*. (Clinical Presentation)	Controls Yes/No (*n*. and Type)	HCM Mean Age, Years (Range or SD)	SVD Score, Methods	SVD Findings	SVD and Fibrosis
Maron et al., 1986 [3]	Autopsy	48 (26 SCD, 9 HF, 10 at operation, 3 others)	Yes (68, including 14 extracardiac and 54 with heart disease increased LV mass)	29 (11–60)	Semiquantitative (mild 1+, moderate 2+, severe 3+)	HCM 83% vs. controls 9%	74% in those with fibrosis vs. 30% in those without
Tanaka et al., 1987 [4]	Autopsy	14 (7 SCD, 1 CVA, 2 others, 4 HF)	Yes (25, hypertensive, cancers and other)	40 (17–76) no-HF 44 (22–60) HF	Quantitative, mean % lumen in the evaluated section (IVS and LV free wall)	HCM: IVS 30%, LV 31% HCM-HF: IVS 17%, LV 33% Hypertensive: IVS 30%, LV 31% Controls: IVS 40%, LV 38%	% lumen inversely correlates with the extent of IVS fibrosis
Basso et al., 2000 [5]	Autopsy	19 (SCD)	Yes (15, trauma)	23 (1–35)	Semiquantitative (0, 1+ <20%, 2+ 20–50%, 3+ >50%), reported as the highest observed score in tissue section	HCM: SVD score 1.2 Controls: no SVD (only 3 hearts with a mean score = 1)	SVD score in areas with and without fibrosis: 1.7 ± 0.4 vs. 1.2 ± 0.4, *p* = 0.04. Positive correlation between %area of fibrosis with SVD mean score (*p* < 0.01, r = 0.58)
Varnava et al., 2000 [16]	Autopsy and HT	72 (22 with HCM-dilated)	No	36 (6–81)	Semiquantitative, % of vessels within a given section in which this ratio between external diameter to lumen diameter is ≥3	% SVD LV: 14.6 (0–50)	No correlation between fibrosis and SVD (r = 0.8, *p* = 0.5), both in total HCM and in HCM-dilated
Kwon et al., 2009 [18]	Myectomy	60 (LVOTO, preserved EF)	No	51(18–76)	Semiquantitative, % of arteries affected by SVD: absent, mild (1–25%), moderate 26–50%) and severe (>50%)	SVD absent 25%, mild 55%, moderate 17%, severe 3%	Septal scar by CMR higher in HCM with SVD than in those without (78% vs. 20%, *p* < 0.001)
Moravsky et al., 2013 [20]	Myectomy	29 (LVOTO, LVEF > 55%)	No	50 (23–77)	Semiquantitative, % of arteries affected by SVD: absent, mild (1–25%), moderate 26–50%), and severe (>50%)	SVD absent 17%, mild 52%, moderate 24%, severe 7%	Statistically significant association between degree of SVD and % replacement fibrosis (*p* = 0.01)
Foà et al., 2019 [6]	Myectomy and HT	57 (27 LVOTO, 30 HF)	No	45.4 (±13.5) LVOTO 46.8 (±12.0) HF	Semiquantitative, lumen stenosis (mild <30%, moderate 30–60%, severe >60%), the highest observed score in tissue section was reported	SVD in 100% LVOTO, severe in 25.9% and multifocal in 55.6% SVD in 93.3% HCM-HF, severe in 30% and multifocal in 70%	No differences between subgroups in terms of SVD, but topographic association between SVD and scar in HCM-HF (70% vs. 40%, *p =* 0.034)
De Gaspari et al., 2021 (current study)	Autopsy and HT	40 (30 SCD, 10 HF)	Yes (20, IHD with HF)	22.6 (7–38) SCD 51.6 (22–68) HF	Semiquantitative (0, 1+ <20%, 2+ 20–50%, 3+ >50–90%, 4 >90%), reported as the highest observed score in tissue section	SVD in 73% HCM-SCD, 100% HCM-HF, 95% IHD SVD score HCM-HF 2.4, HCM-SCD 1.18 and IHD 1.95	SVD score higher in areas with fibrosis vs. those without in both HF-HCM and SCD-HCM hearts (*p* < 0.05)

Abbreviations: CVA, cardiovascular accident; EF, ejection fraction; HF, heart failure; HT, heart transplantation; IHD, ischemic heart disease; IVS, interventricular septum; LV, left ventricle; LVOTO, left ventricular outflow tract obstruction; SCD, sudden cardiac death; SD, standard deviation; SVD, small vessel disease.

## Data Availability

The data presented in this study are available on request from the corresponding author.

## Supplementary Materials

The following are available online at https://www.mdpi.com/2077-0383/10/4/575/s1, Figure S1: Fibrosis and small vessel disease (SVD) prevalence in cases of sudden cardiac death (SCD), heart failure (HF) and ischemic heart disease (IHD), Figure S2: SVD score comparison in males and females (for SCD-HCM, HF-HCM and in the entire HCM population), Table S1: Correlation analysis between age and SVD score (for SCD-HCM and HF-HCM), percentage of mass increase and SVD score (for SCD-HCM and HF-HCM) and percentage of IVS thickness increase and SVD score for SCD-HCM.
