# Supplementary material for: Small Vessel Disease: Another Component of the Hypertrophic Cardiomyopathy Phenotype Not Necessarily Associated with Fibrosis
Source: J Clin Med. 2021 Feb 4;10(4):575. doi: 10.3390/jcm10040575 (PMC7913811; doi:10.3390/jcm10040575)
Supplement: Supplementary file 1 [file jcm-10-00575-s001.pdf]

Journal of
Clinical Medicine

MDPI

Small Vessel Disease: Another Component of the Hypertrophic Cardiomyopathy Phenotype Not Necessarily Associated with Fibrosis

Monica De Gaspari, Cristina Basso *, Martina Perazzolo Marra, Stefania Elia, Maria Bueno Marinas, Annalisa Angelini, Gaetano Thiene and Stefania Rizzo

Cardiovascular Pathology Unit and Cardiology Unit, Azienda Ospedaliera, Department of Cardiac, Thoracic, Vascular Sciences and Public Health, University of Padua Medical School, Via A. Gabelli, 61, 35121 Padova, Italy; monica.deg1@gmail.com (M.D.G.); martina.perazzolomarra@unipd.it (M.P.M.); stefania.e.pd@gmail.com (S.E.); maria.bueno.m@gmail.com (M.B.M.); annalisa.angelini@unipd.it (A.A.); gaetano.thiene@unipd.it (G.T.); s.rizzo@unipd.it (S.R.)

* Correspondence: cristina.basso@unipd.it; Tel.: +39-0498272286

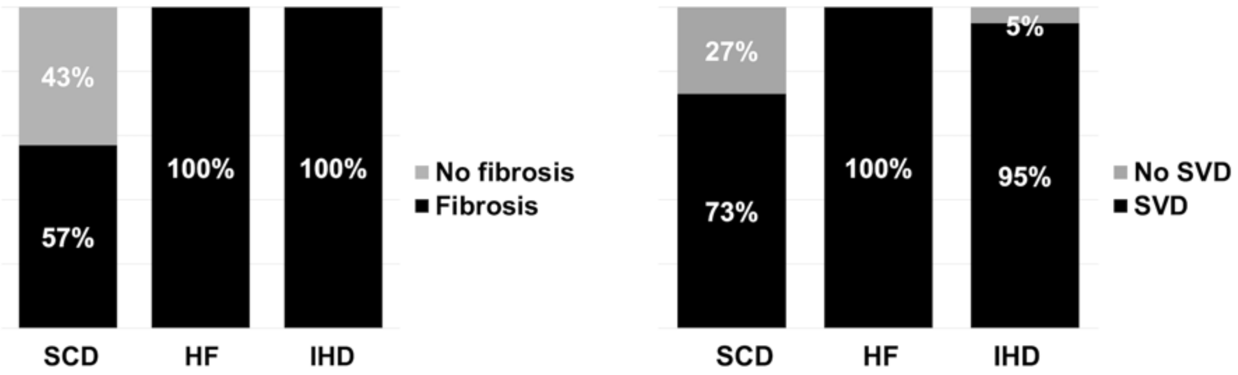

Figure S1. Fibrosis and small vessel disease (SVD) prevalence in cases of sudden cardiac death (SCD), heart failure (HF) and ischemic heart disease (IHD).

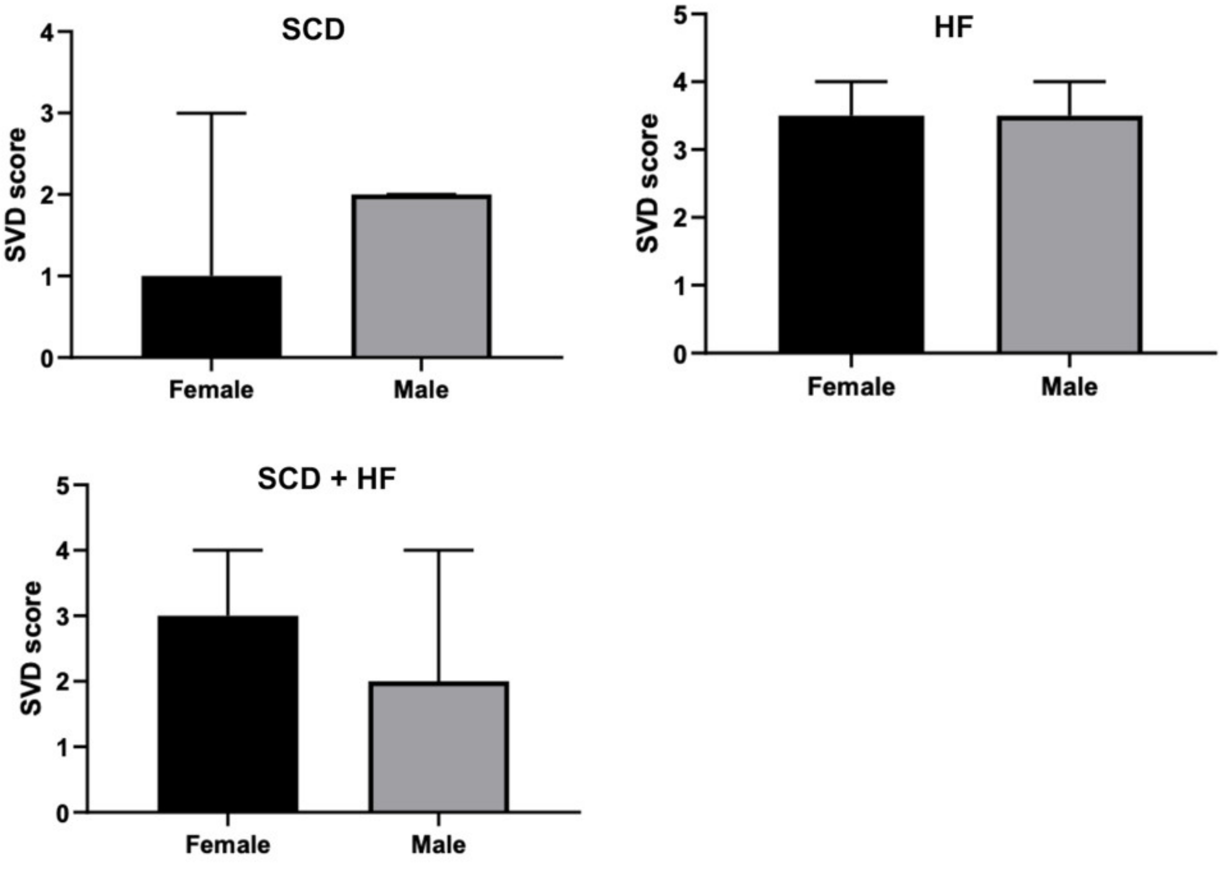

Figure S2. SVD score comparison in males and females (for SCD-HCM, HF-HCM and in the entire HCM population).

Table S1. Correlation analysis between age and SVD score (for SCD-HCM and HF-HCM), percentage of mass increase and SVD score (for SCD-HCM and HF-HCM) and percentage of IVS thickness increase and SVD score for SCD-HCM.

		Correlation Coefficient	*p*-Value
	Age vs SVD score	0.25	0.16
**SCD**	% mass increase vs SVD score	0.38	0.06
	% septal thickness increase vs SVD score	0.32	0.08
**HF**	Age vs SVD score	0.36	0.3
	% mass increase vs SVD score	0.27	0.43
